# Sestrin2 protects against hypoxic nerve injury by regulating mitophagy through SESN2/AMPK pathway

**DOI:** 10.3389/fmolb.2023.1266243

**Published:** 2023-09-21

**Authors:** Cunyao Pan, Chongyi Ai, Lanlan Liang, Baoyi Zhang, Qionglin Li, Lingling Pu, Zirou Wang, Weili Liu, Zhaoli Chen, Hui Liu, Xinxing Wang

**Affiliations:** ^1^ Tianjin Institute of Environmental and Operational Medicine, Tianjin, China; ^2^ School of Public Health, Lanzhou University, Lanzhou, China; ^3^ Chengdu Center for Disease Prevention and Control, Chengdu, China

**Keywords:** hypoxia, mitophagy, SESN2, AMPK, FUNDC1, neurological dysfunction

## Abstract

Hypoxia induced by high altitude can lead to severe neurological dysfunction. Mitophagy is known to play a crucial role in hypoxic nerve injury. However, the regulatory mechanism of mitophagy during this injury remains unclear. Recent studies have highlighted the role of Sestrin2 (SESN2), an evolutionarily conserved stress-inducible protein against acute hypoxia. Our study demonstrated that hypoxia treatment increased SESN2 expression and activated mitophagy in PC12 cells. Furthermore, the knock-out of *Sesn2* gene led to a significant increase in mitochondrial membrane potential and ATP concentrations, which protected the PC12 cells from hypoxic injury. Although the AMPK/mTOR pathway was significantly altered under hypoxia, it does not seem to participate in mitophagy regulation. Instead, our data suggest that the mitophagy receptor FUNDC1 plays a vital role in hypoxia-induced mitophagy. Moreover, SESN2 may function through synergistic regulation with other pathways, such as SESN2/AMPK, to mediate cellular adaptation to hypoxia, including the regulation of mitophagy in neuron cells. Therefore, SESN2 plays a critical role in regulating neural cell response to hypoxia. These findings offer valuable insights into the underlying molecular mechanisms governing the regulation of mitophagy under hypoxia and further highlight the potential of SESN2 as a promising therapeutic target for hypoxic nerve injury.

## 1 Introduction

In recent years, increasing numbers of people, including Europeans and Americans, are traveling to high altitude regions for various purposes including recreation, religion and adventure sports such as mountain climbing in the Everest, Qogir or other high altitude locations around the world (Burtscher, et al., 2021; Mikołajczak, et al., 2021). Despite the inherent risks associated with exposure to high altitude hypoxia, mountaineers are drawn to the breathtaking scenery and the physical and mental challenges presented by these environments. Climbing to high altitudes can lead to exposure to hypoxia, which may result in severe neurological dysfunction such as cognitive impairment, memory loss, and even life-threatening conditions such as high-altitude cerebral edema (HACE) and high-altitude pulmonary edema (HAPE) (Singh and Ansari, 2022). Epidemiological studies and laboratory studies have confirmed that acute exposure to high altitude can contribute to neurological decline (Limmer and Platen, 2018). Mechanistically, declined working memory capacity is closely related to insufficient oxygen supply in the central nervous system. As the “power plant” of cells, mitochondria have a vital function in maintaining neuronal homeostasis in neurons.

Mitophagy is important to remove fragmented or damaged mitochondria and in their quality control (Onishi, et al., 2021). Mitophagy induced by hypoxia mainly exerts a protective function, and is activated principally by autophagy-related proteins (Sulkshane, et al., 2021). Beclin1, LC3, P62 and other conserved proteins participate in the autophagy process and are regarded as autophagy-related proteins (Lv, et al., 2021). Studies have proven that BCL2 interacting protein 3 (BNIP3) and its homolog BCL2 interacting protein 3 like (BNIP3L) belong to the BH3-only protein family and interact directly with microtubule associated protein 1 light chain 3 alpha (LC3) to promote mitophagy (Zhang, et al., 2008). NOD-like receptor (NLR) family member X1 (NLRX1) is suggested to participate in a variety of pathophysiological processes, such as cell death, mitochondrial dynamics, and oxidative damage (Imbeault, et al., 2014; Stokman, et al., 2017; Killackey, et al., 2019). Moreover, recent evidence suggested that FUN14 domain containing 1 (FUNDC1) could serve as a receptor for LC3 and is involved in autophagy (Liu, et al., 2012). The mitophagy receptors harbor LC3-interacting region (LIR) consensus sequences (Ploumi, et al., 2017; Killackey, et al., 2022). Therefore, we hypothesized that BNIP3L, BNIP3, NLRX1, and FUNDC1 function as receptors for selective mitophagy in response to hypoxia via interaction with LC3.

Sestrin2 (SESN2), also known as hypoxia-induced 95 (HI95), is a member of the Sestrin family that has been conserved throughout evolution (Budanov, et al., 2002). SESN2 has been shown to activate adenosine monophosphate-activated protein kinase (AMPK) signaling and downregulate mechanistic target of rapamycin (mTOR) signaling to regulate autophagy (Hou, et al., 2015). In addition, studies have suggested that, as an adaptive response to ischemia-reperfusion (IR), SESN2 plays an essential role in maintaining the function and integrity of mitochondria, thus regulating substrate metabolism (Ren, et al., 2021). However, despite considerable research on SESN2 focusing on hypoxia-ischemia injury and IR injury, the extent to which it regulates hypoxia-induced mitophagy remains unclear (Shi, et al., 2016; Yang, et al., 2017; Quan, et al., 2018). The pathophysiological causes of hypoxic nerve injury vary considerably between IR injury and simple hypoxic injury. Therefore, it is important to investigate the specific role of SESN2 in hypoxia-induced brain injury.

Our study investigated the role of SESN2 in regulating hypoxia-induced mitophagy and neuronal cell adaptation. To this end, we used PC12 cells to investigate the role of SESN2 in the AMPK/mTOR/mitophagy downstream signaling pathway in response to hypoxia-induced injury. We found that hypoxia treatment increased SESN2 expression and activated the mitophagy in PC12 cells. Additionally, our findings suggest that SESN2 may function synergistically with other pathways, such as SESN2/AMPK, to mediate cellular adaptation to hypoxia, potentially making it a therapeutic target for hypoxic nerve injury.

## 2 Materials and methods

### 2.1 Culture of cells and modeling of hypoxia

PC12 cells (BFN60070191) were grown in a 5% CO_2_ atmosphere at 37°C in Dulbecco’s modified Eagle’s medium (DMEM [Sigma-Aldrich Corporation, D6429]) containing 10% fetal bovine serum [FBS (Atlanta Biologicals, S12450)], 2 mmol L-glutamine (GIBCO, 25,030-081), and 1% penicillin/streptomycin (Life Technologies, 15140163). Cells to be treated with hypoxia were placed in a tri-gas incubator (InvivO2, I400) at 37°C under 5% CO_2_ and 0.5% O_2_. The incubator could control the oxygen conc from 0.1% to 23.0% by regulating the ratio of N_2_/O_2_. The Subsequent steps would continue in this incubator if needed.

### 2.2 Knockout of *Sesn2* using a lentivirus-delivered CRISPR/cas9 system

A *Sesn2*-knockout cell line was generated using the CRISPER/Cas9 system (Shanghai Jikai Gene Chemical Technology Co., Ltd). Green fluorescent protein (GFP)-LC3 (Shanghai Jikai Gene Chemical Technology Co., Ltd.) was used to assess autophagic flux by an inverted light microscope (Leica DMi8; Leica Microsystems, Wetzlar, Germany). Stable cell lines were produced by selection with 2.5 μg/mL puromycin (Sigma).

### 2.3 Cell viability detection

1A Cell Counting Kit-8 [CCK-8 (Biosharp, BS350C)] was used to test cell viability. Briefly, cells were seeded at 8,000 cells per well into 96-well plates. Then, the cells were exposed to hypoxia, followed by the addition of 100 μL fresh medium with 10 μL of CCK-8 reagent and incubated for 30 min at 37°C. The absorbance of the cells at 450 nm was assessed using a Microplate Reader (M5; Molecular Devices, San Jose, CA, United States).

### 2.4 Determination of cell reactive oxygen species (ROS)

A ROS assay kit (Beyotime, S0033M) was applied to measure ROS levels. Briefly, PC12 cells were added with 10 μM 2,7-dichlorodihydrofluorescein diacetate (DCFH-DA) and incubated for 30 min. Thereafter, the DCFH-DA was removed by rinsing the cells two times using Phosphate-buffered saline [PBS (Procell, PB180327)]. The cells were then digested using pancreatin, resuspended in PBS, and subjected to flow cytometry (BD Biosciences, San Jose, CA, United States) to detect the ROS levels.

### 2.5 Cell apoptosis assay

Flow cytometry was used to determine cell apoptosis. Collected cells were rinsed using PBS and incubated in the presence of propidium iodide (PI) and annexin V-fluorescein isothiocyanate (FITC) (BD Biosciences, 556,547) for 15 min at room temperature in the dark. Flow cytometry was then used to determine cell apoptosis following the supplier’s protocols.

### 2.6 Determination of the mitochondrial membrane potential (MMP)

The MMP was detected using the fluorescent probe JC-1 (Beyotime, C2005). Briefly, 1 mL of JC-1 working solution was added to PC12 cells and incubated for 20 min at 37°C, washed two times using JC-1 buffer, and then subjected to flow cytometry analysis.

### 2.7 ATP content determination

Cells were collected into pre-cooled PBS, frozen quickly as aliquots, and stored in liquid nitrogen. For use, aliquots were allowed to melt slowly in an ice water bath and vortexed for 10 s. The ATP content was assayed via Luciferase driven bioluminescence using an ATP Bioluminescence Assay Kit HS II (Sigma-Aldrich, 11699709001). The relative light units of each sample were detected with Microplate Reader (M5; Molecular Devices, San Jose, CA, United States).

### 2.8 Western blotting

Western blotting was performed according to a standard protocol. The cell lysates (20 μg/lane) were separated using 4–12% or 15% SDS-PAGE gel and then transferred to nitrocellulose membranes. The membrane was blocked with 5% skim milk diluted in TBST, and further incubated with primary antibodies overnight at 4°C. Primary antibodies to the following proteins were used: SESN2 (1:1,000, Cell Signaling Technology, 8,487), AMPK (1:1,000, Cell Signaling Technology, 5,832), phosphorylated (p)-AMPK (1:1,000, Cell Signaling Technology, 2,535), mTOR (1:1,000, Cell Signaling Technology, 2,983), p-mTOR (1:1,000, Cell Signaling Technology, 2,974), Beclin1 (1:1,000, Cell Signaling Technology, 3,495), P62 (1:1,000, Cell Signaling Technology, 23,214), LC3B (1:1,000, Cell Signaling Technology, 2,775), translocase of outer mitochondrial membrane 20 (Tomm20) (1:1,000, Cell Signaling Technology, 42,406), BNIP3L (1:1,000, Cell Signaling Technology, 12,396), BNIP3 (1:1,000, Cell Signaling Technology, 3,769), FUNDC1 (1:1,000, Cell Signaling Technology, 49,240), NLRX1 (1:1,000, Cell Signaling Technology, 13,829), and β-actin (1:50,000, Abclonal, AC038). Secondary antibodies to the following proteins were used: Goat Anti-Rabbit HRP (1:10,000, Abclonal, AS014). Membrane stripping was performed by incubating the membrane in Stripping Buffer (CWBIO, Stripping Buffer, CW0056M) according to the manufacturer’s instructions. The immunoreactive protein bands were exposed by the enhanced chemiluminescence (ECL) method (General Electric, Boston, MA, United States) and normalized by densitometry using ImageJ (NIH, Bethesda, MD, United States).

### 2.9 Quantitative real-time reverse transcription PCR (qRT-PCR)

The Trizol reagent (Invitrogen, 15596026) was used to extract total RNA following the manufacturer’s protocol. The mRNA was reverse transcribed to cDNA. The cDNA was then used as the template in the quantitative real-time PCR (qPCR) step, which was carried out using a SYBR Green Real-time PCR Master Mix (Thermo Fisher Scientific, A46109), and *Actb* (encoding β-actin) as the internal control. The 2^−ΔΔCT^ method was used to analyze the results (Livak and Schmittgen, 2001). The primers used had the following sequences:

Bnip3l forward, 5′-TCT​CAC​TTA​GTC​GAG​CCG​C-3′ and reverse 5′-CTC​CAC​CCA​GGA​ACT​GTT​GA-3’; *Bnip3* forward, 5′-TCT​CAC​TTA​GTC​GAG​CCG​C-3′ and reverse 5′-CTC​CAC​CCA​GGA​ACT​GTT​GA-3’; *Fundc1* forward, 5′-TCT​CAC​TTA​GTC​GAG​CCG​C-3′ and reverse 5′-CTC​CAC​CCA​GGA​ACT​GTT​GA-3’; *Nlrx1* forward, 5′-TCT​CAC​TTA​GTC​GAG​CCG​C-3′ and reverse 5′-CTC​CAC​CCA​GGA​ACT​GTT​GA-3′

### 2.10 Statistical analysis

The statistical analyses were carried out using GraphPad Prism (GraphPad Inc., V5.0.1). Student’s t-test was used to calculate the difference between two groups, whereas analysis of variance (ANOVA) followed by the Tukey-Kramer multiple comparisons test or an unpaired two-tailed Student-t-test were used to analyze three and more groups of data. All data are shown as mean ± SD for each group. *p* < 0.05 was considered statistically significant.

## 3 Results

### 3.1 Hypoxia induced PC12 cell injury and increased expression of SESN2

**FIGURE 1:**
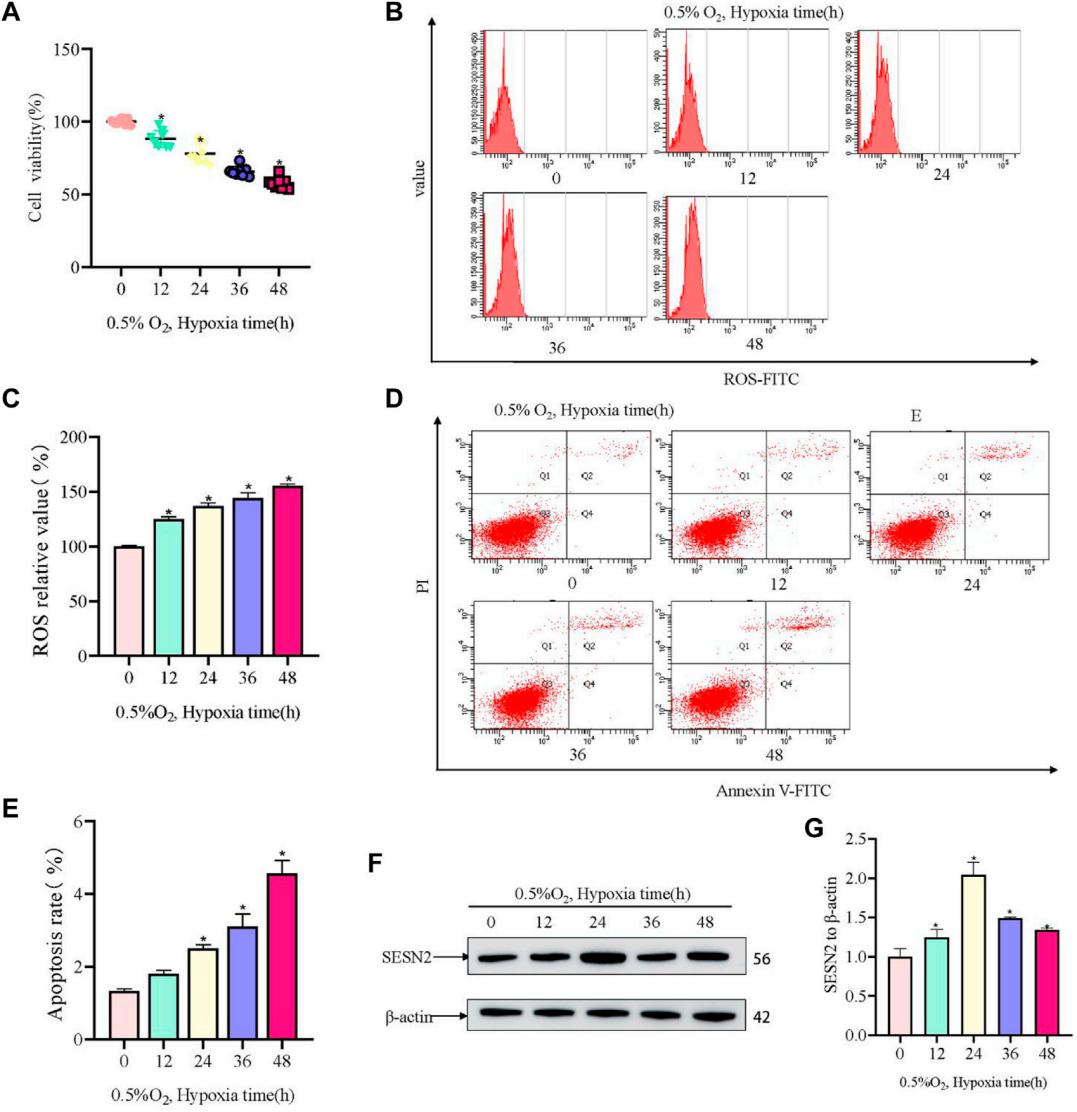
Hypoxia led to decreased cellular activity, elevated ROS levels, and elevated apoptosis rates in PC12 cells. **(A)** Cellular activity over hypoxia time in PC12 cells (0, 12, 24, 36, and 48 h at 0.5% O_2_). **(B)** ROS levels over hypoxia time in PC12 cells. **(C)** Quantitative analysis of **(B)**. **(D)** Apoptosis rate over hypoxia time in PC12 cells. **(E)** Quantitative analysis of **(D)**. **(F)** Protein levels of SESN2 over hypoxia time in PC12 cells (0, 12, 24, 36, and 48 h at 0.5% O_2_). **(G)** Quantitative analysis of **(F)**. Data are presented as mean ± SD. * indicates a significant increase compared with the control value (*p* < 0.05, *n* = 3).

Firstly, a hypoxia-induced model of PC12 cell damage was constructed. In the model, hypoxia induced injury to PC12 cells in a time-dependent manner. Hypoxic (0.5% O_2_) conditions for 12 h resulted in significant injury to PC12 cells, with the OD450 value in the CCK8 assay (representing cell viability) decreasing by about 50% after 48 h (Figure 1A). Hypoxia increased ROS activity in PC12 cells, and the ROS level correlated with increased hypoxia time (Figures 1B, C). The apoptotic rate of PC12 cells increased with hypoxia time in culture (Figures 1D, E). Furthermore, western blotting showed that the level of SESN2 increased at first and then decreased, with the highest levels being observed at 24 h (Figures 1F, G). Thus, we hypothesized that SESN2 might be involved in response to the duration of hypoxia.

### 3.2 Sesn2 knockout could protect mitochondrial function in hypoxia-damaged PC12 cells

**FIGURE 2:**
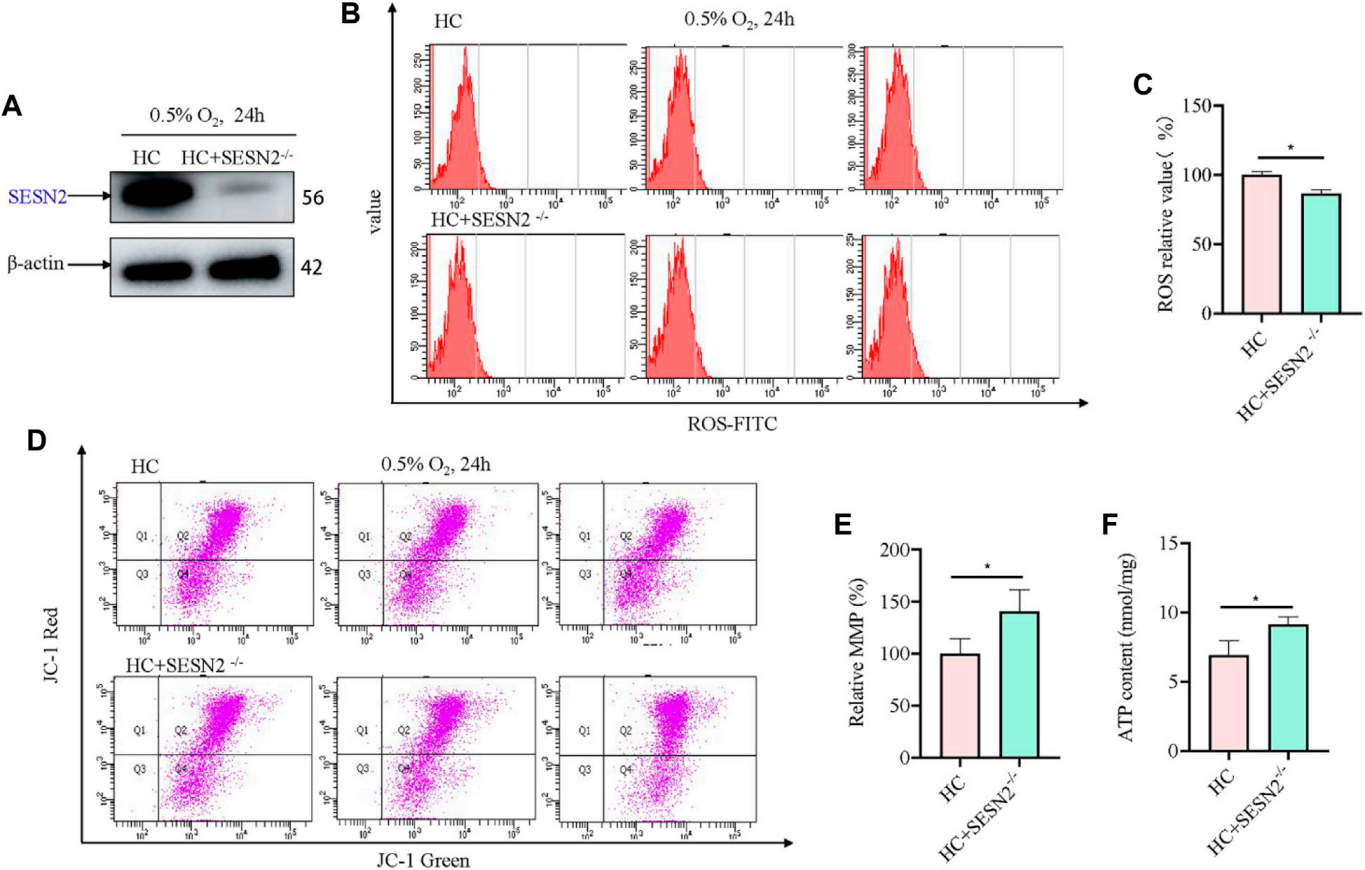
*Sesn2* knockout improved mitochondrial function of PC12 cells under hypoxia. **(A)** Effect of *Sesn2* knockout. **(B)** Knockout of *Sesn2* decreased intracellular ROS levels of PC12 cells under hypoxic conditions for 24 h. 3 replicate data of HC and HC + SESN2^−/−^ group was shown respectively. **(C)** Quantitative analysis of **(B)**. **(D)** Knockout of *Sesn2* increased mitochondria membrane potential in PC12 cells under hypoxic conditions for 24 h. **(E)** Quantitative analysis of **(D)**. **(F)** Knockout of *Sesn2* upregulated the mitochondrial ATP content in PC12 cells under in hypoxic conditions for 24 h. HC: Hypoxia Control. Data are presented as mean ± SD. * indicates a significant increase compared with the control value (*p* < 0.05, *n* = 3).

Based on above results, to investigate the role of SESN2 in hypoxic metabolism, the *Sesn2* gene was knocked out using the CRISPR/cas9 system, and a stable *Sesn2* ^−/−^ PC12 cell line was constructed, which was screened using puromycin for 5–7 days. Western blotting of proteins extracted from *Sesn2* ^−/−^ PC12 cells showed the almost complete absence of SESN2 (Figure 2A). To determine the effect of *Sesn2* knockout on mitochondrial function, we determined the ROS and ATP levels, and the incorporation of JC-1 to assess the mitochondrial membrane potential (MMP), in response to hypoxia 24 h. The results showed knockout of *Sesn2* reduced ROS levels under hypoxic conditions compared with those under normal conditions (Figure 2B, C). The mitochondrial membrane potential and mitochondrial ATP levels were increased after *Sesn2* knockout under hypoxic conditions compared with those under normal conditions (Figures 2D–F).

### 3.3 Mitophagy is involved in the regulation of hypoxic metabolism in PC12 cells

**FIGURE 3:**
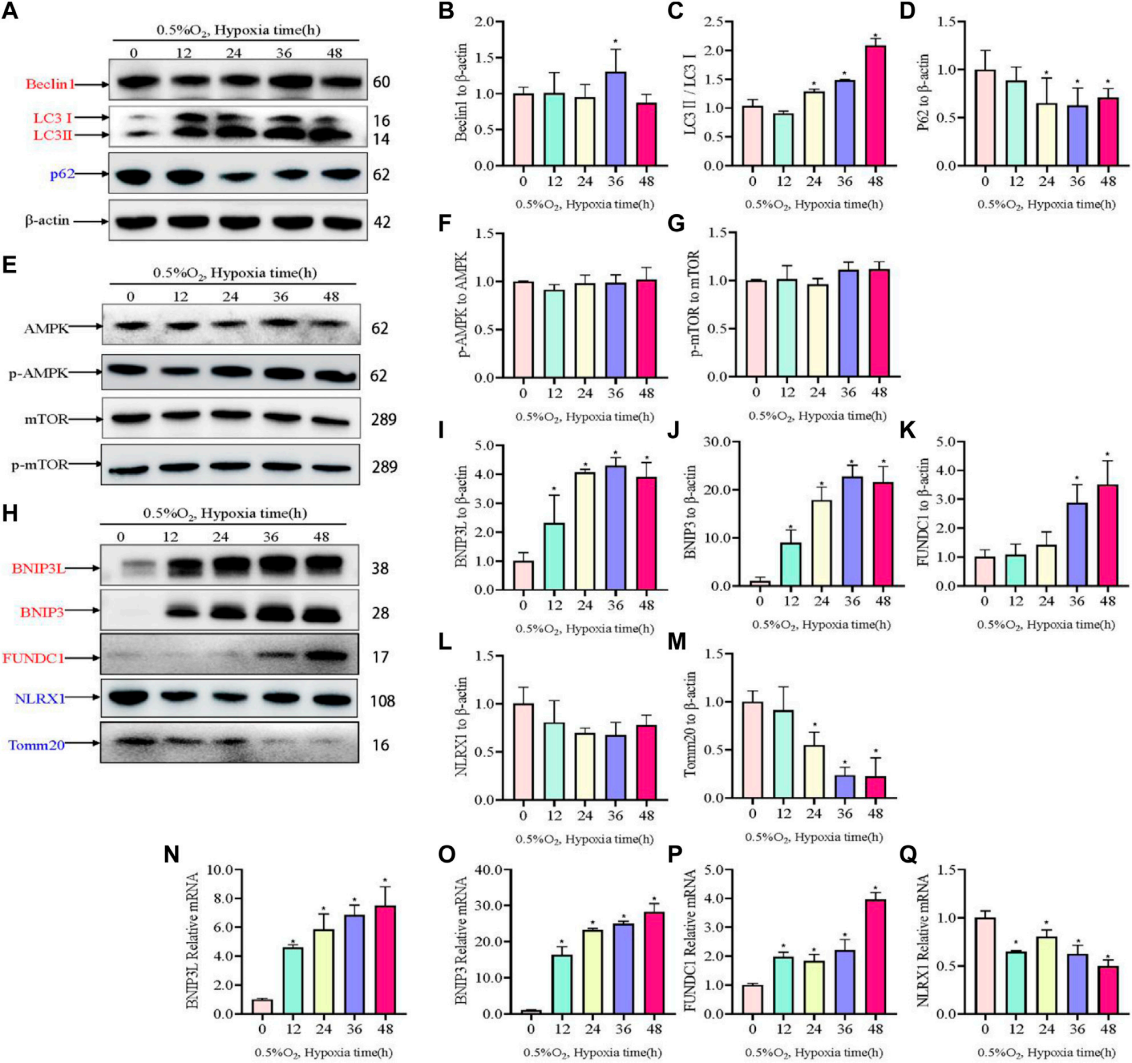
Levels of autophagy-related proteins under hypoxia in PC12 cells. **(A)** Protein levels of Beclin1, LC3 I, LC3 II, and p62 over hypoxia time in PC12 cells (0, 12, 24, 36, and 48 h at 0.5% O_2_). **(B–D)** Quantitative analysis of **(A)**. **(E)** AMPK, p-AMPK, mTOR and p-mTOR levels over hypoxia time in PC12 cells. **(F–G)** Quantitative analysis of p-AMPK-AMPK and p-mTOR-mTOR. **(H)** Protein levels of BNIP3L, BNIP3, FUNDC1, NLRX1, and Tomm20 over hypoxia time in PC12 cells. **(I–M)** Quantitative analysis of **(H)**. **(N–Q)** mRNA expression of *Bnip3l*, *Bnip3*, *Fundc1*, and *Nlrx1* over hypoxia time. Blue indicates a decrease in the protein level and red indicates an increase in the protein level. Data are presented as mean ± SD. * indicates a significant increase compared with the control value (*p* < 0.05, *n* = 3).

Hypoxic injury involves damage to mitochondria; therefore, to assess to changes to mitochondria and autophagosomes, the levels of autophagosome-associated proteins and mitophagy receptors were assessed using western blotting. Hypoxia increased the levels of the autophagy proteins Beclin1 and LC3II/I, but reduced the expression of P62 under hypoxic conditions compared with those under normal conditions (Figures 3A–D). The Sestrin2/AMPK/mTOR pathway has been well-established as a critical regulator of autophagy; therefore, the levels of AMPK, p-AMPK, mTOR, and p-mTOR were determined. Somewhat to our surprise, there was no change in the level of p-AMPK and p-mTOR in response to hypoxia (Figures 3E–G). Moreover, we evaluated changes in mitophagy protein markers. Hypoxia increased the levels of BNIP3L, BNIP3, and FUNDC1, but decreased the levels of NLRX1 (Figure 3H–L). TOMM20, which served as a mitochondrial marker, decreased in a time dependent manner in response to hypoxia. Concomitantly, the expression levels of mitophagy-related genes were investigated using qRT-PCR, the results of which were consistent with those of western blotting (Figure 3M–Q).

### 3.4 Inhibition of autophagy by 3-methyladenine (3 MA) also improved mitochondrial function after hypoxia

**FIGURE 4:**
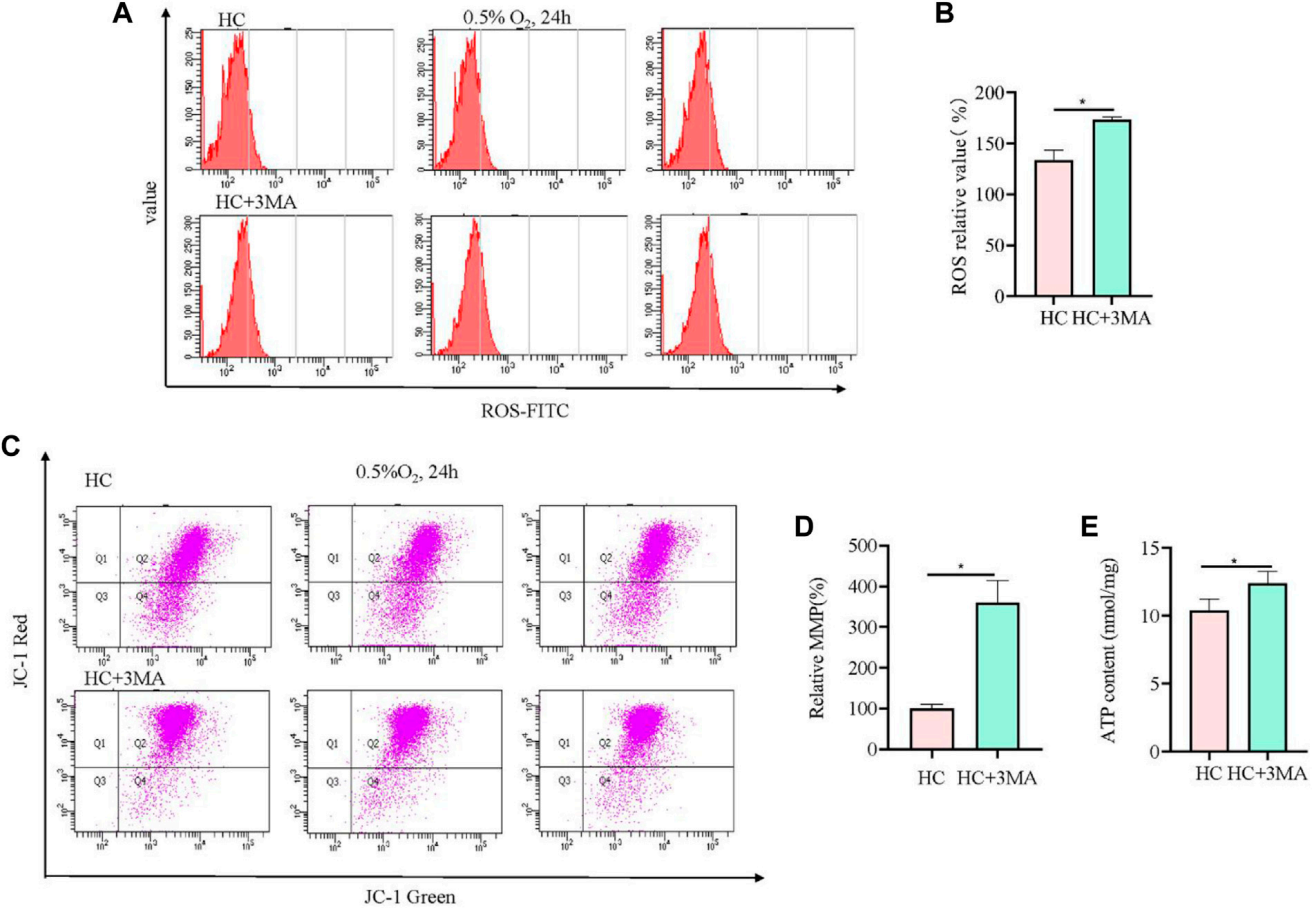
3MA led to elevated ROS levels, increased mitochondrial membrane potential, and upregulated mitochondrial ATP content. **(A)** In PC12 cells subjected to hypoxia (0.5% O_2_, 24 h), 3MA increased intracellular ROS. 3 replicate data of HC and HC + 3MA group was shown respectively. **(B)** Quantitative analysis of **(A)**. **(C)** In PC12 cells subjected to hypoxia (0.5% O_2_, 24 h), 3 MA increased the mitochondrial membrane potential. **(D)** Quantitative analysis of **(C)**. **(E)** In PC12 cells subjected to hypoxia (0.5% O_2_, 24 h), 3 MA upregulated the mitochondrial ATP content. HC: Hypoxia Control. Data are presented as mean ± SD. * indicates a significant increase compared with the control value (*p* < 0.05, *n* = 3).

To further validate the role of mitophagy in hypoxia-injured neural cells, the autophagy inhibitor 3 MA was used to pretreat cells at 5 mM for 12 h. Compared with *Sesn2*-knockout cells, the analysis of ROS showed the opposite results, i.e., 3 MA pre-treatment caused elevation of ROS levels (Figure 4A, B). The results from the JC-1 and ATP experiments showed the same trend to those obtained in the *Sesn2*-knockout cells, indicating the protective effect of autophagy inhibition on mitochondrial repair in response to hypoxia (Figures 4C–E).

### 3.5 Sesn2 knockout significantly inhibited p-AMPK levels and mitophagy processes after hypoxia

**FIGURE 5:**
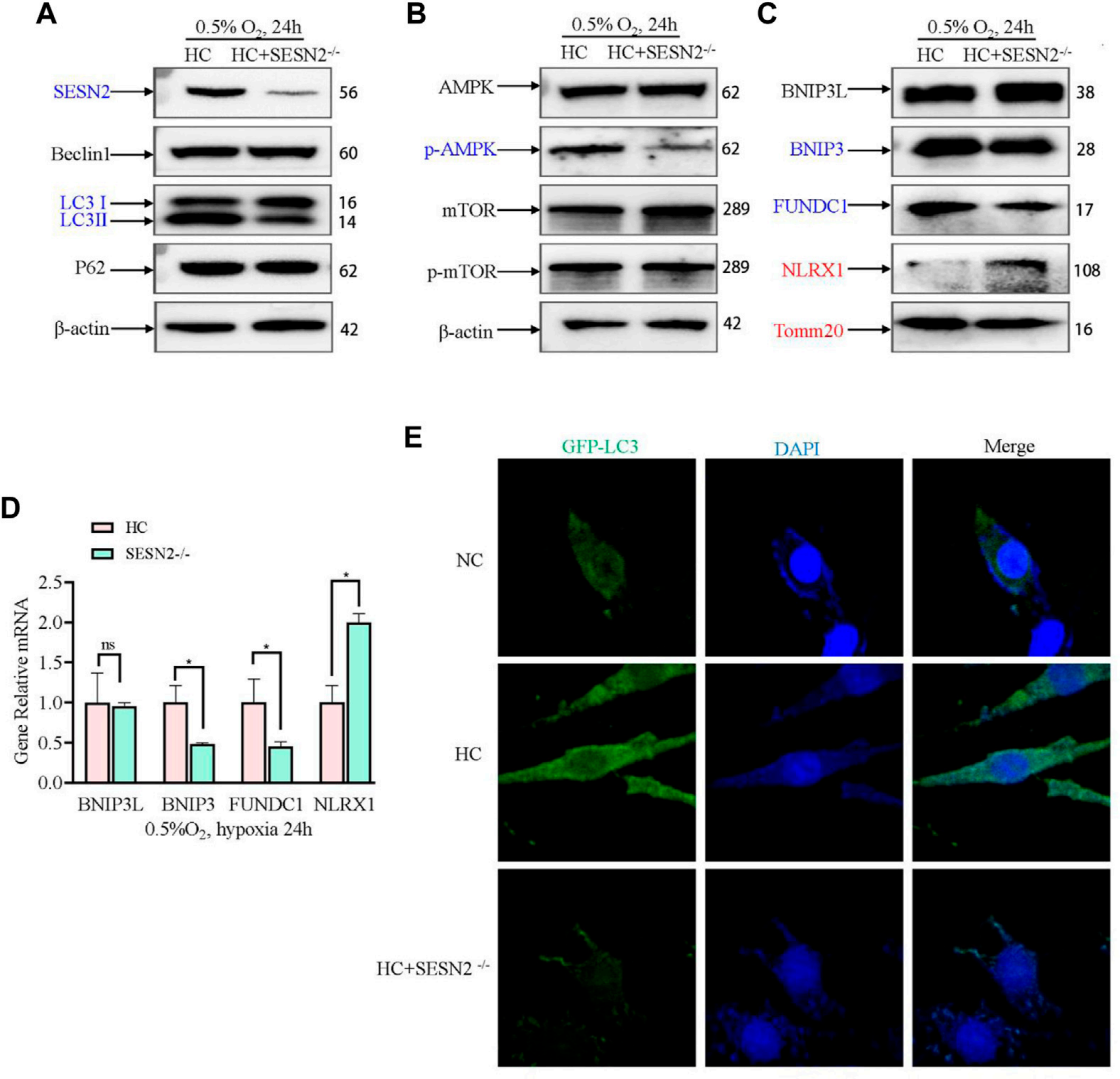
Effect of SESN2 knockout on mitophagy-related proteins under hypoxia in PC12 cells. **(A)** Levels of Beclin1, LC3 I, LC3 II, and p62 proteins were determined after *Sesn2* knockout using western blotting in PC12 cells subjected to hypoxia (0.5% O_2_, 24 h). **(B)** AMPK, p-AMPK, mTOR and p-mTOR levels after *Sesn2* knockout in PC12 cells subjected to hypoxia (0.5% O_2_, 24 h). **(C)** Protein levels of BNIP3L, BNIP3, FUNDC1, NLRX1, and Tomm20 after *Sesn2* knockout in PC12 cells (0.5% O_2_, 24 h). **(D)** mRNA expression of *Bnip3l*, *Bnip3*, *Fundc1*, and *Nlrx1* after knockout in PC12 cells subjected to hypoxia (0.5% O_2_, 24 h). Data are presented as mean ± SD. * indicates a significant increase compared with the control value (*p* < 0.05, *n* = 3). **(E)** Detection of GFP-LC3 autophagosomes after *Sesn2* knockout in PC12 cells subjected to hypoxia (0.5% O_2_, 24 h, magnification: ×400). HC: Hypoxia Control. NC: Normoxia Control. Blue indicates a decrease in the protein level and red indicates an increase in the protein level.

Next, in view of its role in hypoxia regulation, AMPK-mediated mitophagy was investigated to further study the mechanism by which *Sesn2* knockout protected mitochondria against hypoxic injury. *Sesn2* knockout did not affect Beclin1 and P62 levels significantly, whereas the level of LC3II/I decreased (Figure 5A). Moreover, *Sesn2* knockout decreased the p-AMPK:AMPK ratio significantly, but had no effect on the p-mTOR: mTOR ratio (Figure 5B). The expression of mitophagy proteins and mitochondrial outer membrane proteins were measured using western blotting and qRT-PCR. We found that the mRNA and protein levels of BNIP3 and FUNDC1 decreased significantly, and NLRX1 and TOMM20 mRNA and protein levels increased significantly after *Sesn2*-knockout (Figure 5C, D). Concomitantly, transfection with GFP-LC3 was employed to assess autophagic flux, which confirmed the above results: Hypoxia caused mitophagy and *Sesn2*-knockout inhibited autophagy activation (Figure 5E).

### 3.6 Mitophagy was inhibited by 3 MA but did not alter SESN2 expression

**FIGURE 6:**
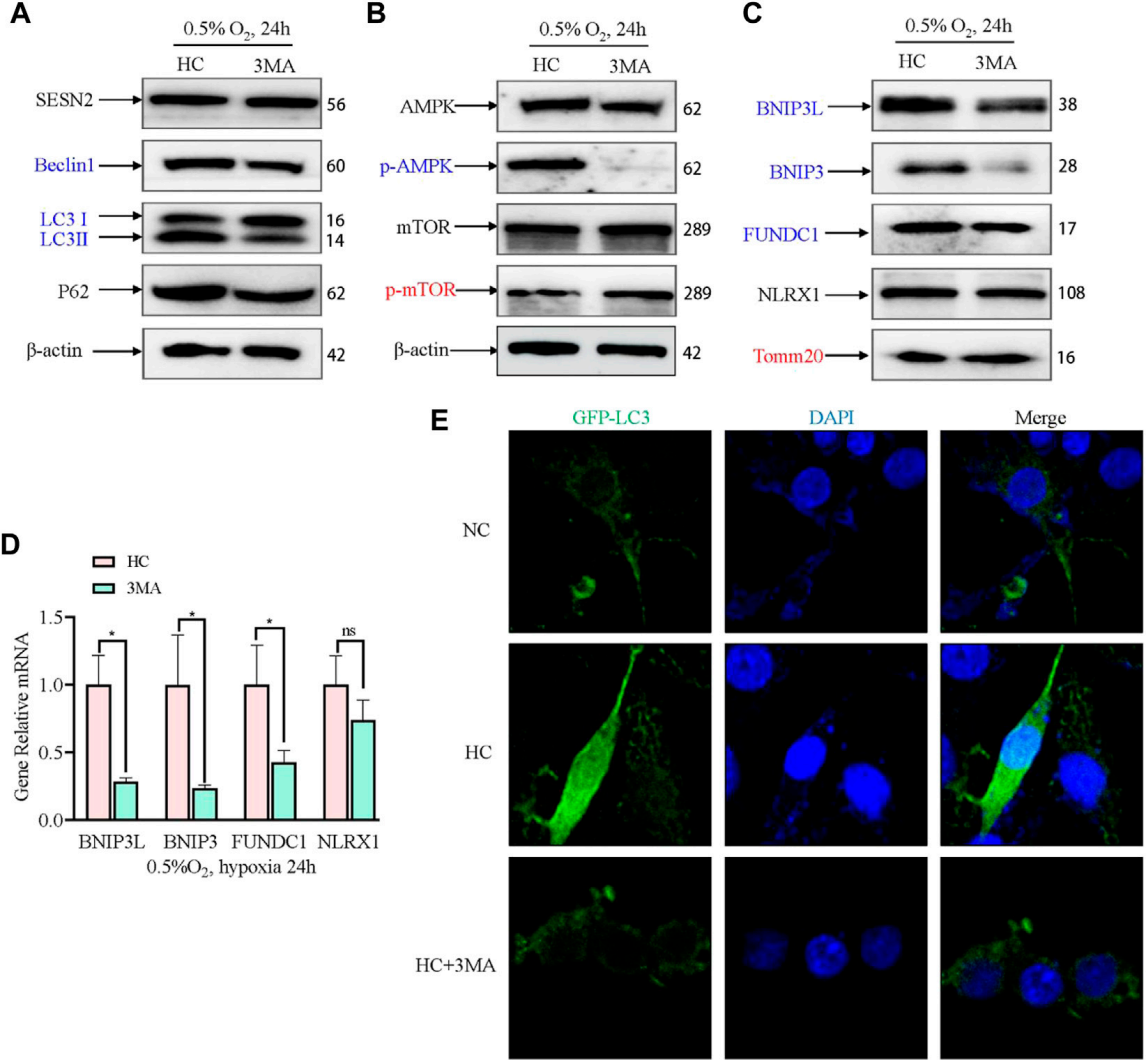
Effect of 3 MA on mitophagy-related proteins under hypoxia in PC12 cells. **(A)** Effects of 3 MA on the protein expression of SESN2, Beclin1, LC3B, and P62 proteins by western blotting in PC12 cells subjected to hypoxia (0.5% O_2_, 24 h). **(B)** Effects of 3 MA on p-mTOR, mTOR, p-AMPK, and AMPK levels in PC12 cells subjected to hypoxia (0.5% O_2_, 24 h). **(C)** Effects of 3 MA on the protein levels of BNIP3L, BNIP3, FUNDC1, NLRX1, and Tomm20 in PC12 cells subjected to hypoxia (0.5% O_2_, 24 h). **(D)** mRNA expression of *Bnip3l*, *Bnip3*, *Fundc1*, and *Nlrx1* after 3 MA intervention in PC 12 cells subjected to hypoxia (0.5% O_2_, 24 h). Data are presented as mean ± SD. * indicates a significant increase compared with the control value (*p* < 0.05, *n* = 3). **(E)** Detection of GFP-LC3 autophagosomes after 3 MA intervention in PC12 cells subjected to hypoxia (0.5% O_2_, 24 h, magnification: ×400). HC: Hypoxia Control. NC: Normoxia Control. Blue indicates a decrease in the protein level and red indicates an increase in the protein level.

To confirm that the expression of SESN2 mediates mitophagy by autophagy activation, we used an autophagy inhibitor (3 MA) to block the initiation of autophagy. The AMPK signaling pathway and mitophagy proteins were then detected. 3MA reduced the level of Beclin1 and LC3B, but exhibited no appreciable effect on the levels of SESN2 and P62 (Figure 6A). 3MA activated mTOR by inhibiting AMPK (Figure 6B). Furthermore, 3 MA treatment significantly decreased the BNIP3L, BNIP3, and FUNDC1 levels, increased the level of TOMM20, and had no effect on NLRX1 (Figure 6C, D). Consistent with *Sesn2* knockdown, the addition of 3 MA also decreased the numbers of GFP-LC3–positive cells after hypoxia treatment (Figure 6E).

## 4 Discussion

**FIGURE 7:**
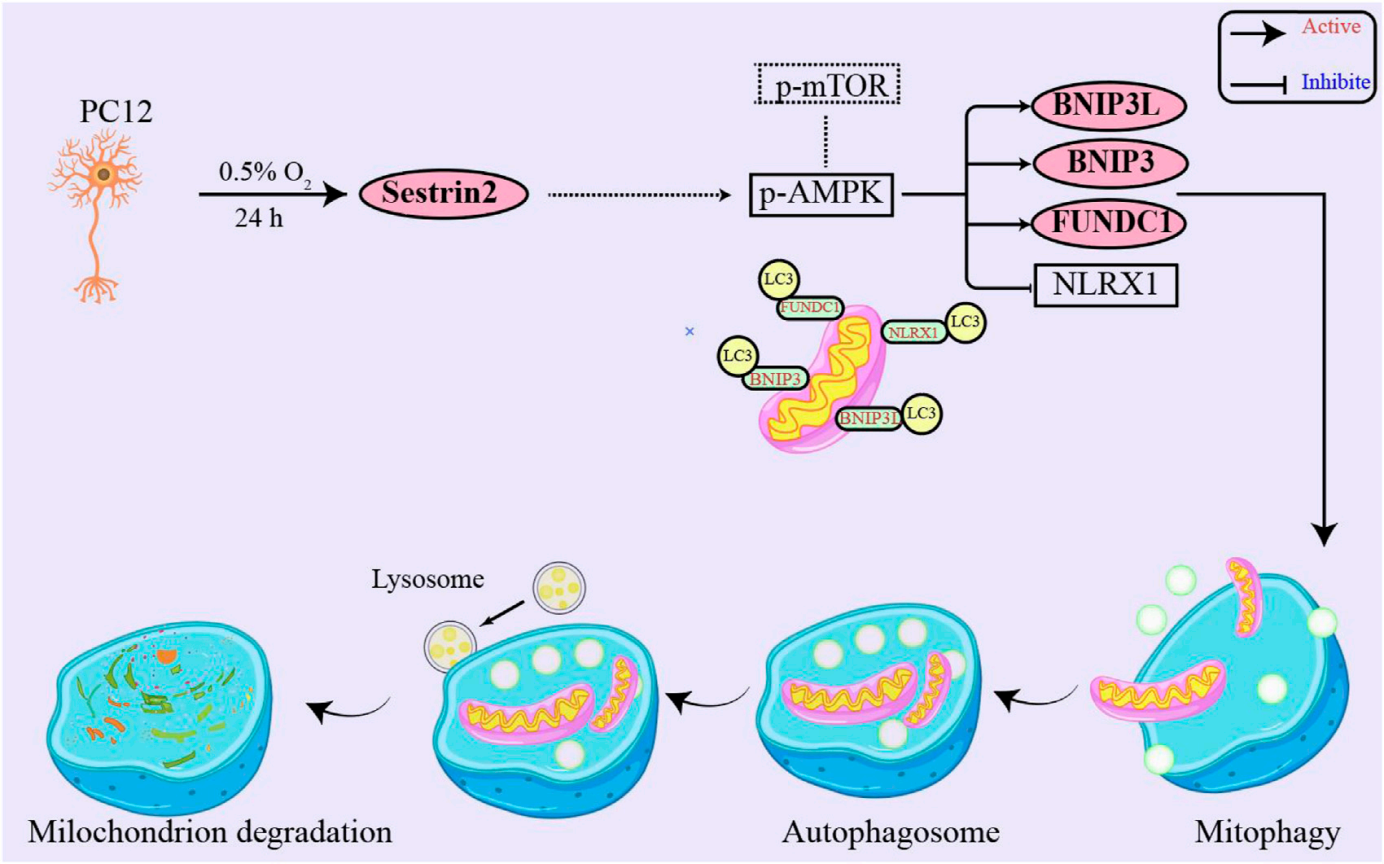
SESN2 upregulation activated mitophagy in PC12 cells by hypoxia treatment. Mitophagy receptor FUNDC1 plays a vital role in hypoxia-induced mitophagy. Moreover, SESN2 upregulation may function through synergistic regulation with other pathways, such as SESN2/AMPK, to mediate cellular adaptation to hypoxia. Therefore, SESN2 plays a critical role in regulating neural cell response to hypoxia.

SESN2 is a stress-responsive protein that has been shown to respond to various insults, such as oxidative stress, genotoxic stress, energy deficiency, and hypoxia (Budanov, et al., 2002; Pan, et al., 2021). While the role of SESN2 has been extensively studied in I/R-related diseases, its effect on high altitude hypoxia has been rarely reported (Shi, et al., 2016; Wang, et al., 2019a; Wang, et al., 2019b). The present study aimed to investigate whether SESN2 plays a role in the hypoxic response and to elucidate its possible mechanisms. Using *in vitro* studies, we found that SESN2 promotes mitophagy and reduces the mitochondrial quantity of hypoxic neuroblasts. Our results suggest that the SESN2/AMPK/mTOR/FUNDC1 signaling pathway regulates the process of mitophagy, as shown in Figure 7. These findings provide new insights into the molecular mechanisms underlying hypoxia-induced neuronal injury and highlight SESN2 as a promising therapeutic target for treating cognitive impairment caused by high altitude. In our experiments, we observed significant upregulation of SESN2 expression following hypoxia treatment, consistent with previous reports investigating hypoxia-ischemia (Shi, et al., 2016) and ischemia-reperfusion (Liu, et al., 2020).

To assess autophagic flux, we measured the levels of LC3-II, Beclin1, and p62 proteins, which are indicators of autophagy. Our results indicated that hypoxia treatment activated autophagy and increased autophagic flux. Autophagy can be triggered by either mTOR downregulation or AMPK activation (Egan, et al., 2011). However, our findings suggested that hypoxia-induced autophagy flux is not dependent on the AMPK/mTOR pathway. Degradation of damaged or dysfunctional mitochondria involves mitophagy, a specific form of autophagy (Ashrafi and Schwarz, 2013). Mitophagy receptors such as BNIP3L, BNIP3, FUNDC1, and NLRX1 recruit LC3 to initiate mitophagy in response to mitochondrial stress (Rouschop and Wouters, 2009; Fang, et al., 2015), including oxidative stress, bioenergetic stress, and IR stress (Liu, et al., 2012; Ney, 2015). The different mitophagy mechanisms that sense these stresses are still unclear. Our results suggest that BNIP3L, BNIP3, FUNDC1, and NLRX1 are involved in hypoxia-induced mitophagy.

To determine the role of SESN2 in neurocytes under hypoxia, we knocked out *Sesn2* in PC12 cells. We observed that *Sesn2* knockout increased ROS and ATP levels and mitochondrial membrane potential. Mechanistically, *Sesn2* knockout significantly reduced AMPK phosphorylation, inhibited mitophagy, and protected PC12 cells. *Sesn2* knockout also suppressed BNIP3 and FUNDC1 expression, while increasing NLRX1 protein. Treatment with 3MA, an autophagy inhibitor, effectively suppressed hypoxia-induced mitophagy, resulting in increased mitochondrial membrane potential and ATP production without any notable effect on SESN2 protein levels. Therefore, we conclude that SESN2 acts upstream of the autophagic cascade. Our experiments further suggest that *Sesn2* knockout repairs mitochondrial function by inhibiting AMPK-mediated mitophagy.

Previous studies have indicated that the SESN2/AMPK/mTOR pathway plays a dominant role in mediating autophagy in hypoxia-related diseases (Hou, et al., 2015; Hwang, et al., 2017). To test whether SESN2 regulates AMPK and its downstream mitophagy receptor, we carried out a series of experiments. *Sesn2* knockout suppressed AMPK activation, while upregulation of SESN2 contributed to AMPK phosphorylation. Further, *Sesn2* knockout significantly reduced BNIP3 and FUNDC1 expression, but NLRX1 protein was decreased too. Zhang et al. (2019a) identified NLRX1 as a novel mitophagy receptor that induces mitophagy, and Li et al. (2021) demonstrated that intestinal I/R injury downregulates NLRX1 levels, consistent with our findings. The pathophysiological role of BNIP3 in our experiments remains unknown, though most studies have reported that it can induce apoptosis (Prabhakaran, et al., 2007; Jia, et al., 2020) while Zhang et al. revealed that BNIP3 could promote mitophagy (Zhang, et al., 2019b).

In summary, our study provides valuable insights into the role of SESN2 in hypoxia-induced nerve injury and its potential as a therapeutic target. Hypoxia treatment increases SESN2 expression, while *Sesn2* knockout leads to reduced AMPK activation and mitophagy signaling. These observations highlight the critical role of the SESN2/AMPK pathway in regulating hypoxia-induced autophagy to exert neuroprotective effects and provide a deeper understanding of the role of SESN2 in regulating hypoxia-induced autophagy. Hence, our study reveals a previously unexplored role for SESN2 as a therapeutic intervention for hypoxic nerve injury. Furthermore, targeting SESN2 may hold promise in regulating the response to high altitude hypoxia. However, further research is required to confirm these findings and fully understand the underlying pathways involved.

## Data Availability

The original contributions presented in the study are included in the article/Supplementary Material, further inquiries can be directed to the corresponding authors.
