# Corrigendum: Sestrin2 protects against hypoxic nerve injury by regulating mitophagy through SESN2/AMPK pathway

**DOI:** 10.3389/fmolb.2024.1392150

**Published:** 2024-04-05

**Authors:** Cunyao Pan, Chongyi Ai, Lanlan Liang, Baoyi Zhang, Qionglin Li, Lingling Pu, Zirou Wang, Weili Liu, Zhaoli Chen, Hui Liu, Xinxing Wang

**Affiliations:** ^1^ Tianjin Institute of Environmental and Operational Medicine, Tianjin, China; ^2^ School of Public Health, Lanzhou University, Lanzhou, China; ^3^ Chengdu Center for Disease Prevention and Control, Chengdu, China

**Keywords:** hypoxia, mitophagy, SESN2, AMPK, FUNDC1, neurological dysfunction

In the published article, there was an error in the **Funding** statement. The funding statement was displayed as “The author(s) declare that no financial support was received for the research, authorship, and/or publication of this article.” The correct **Funding** statement appears below.

