# Supplementary figures and images for: Sestrin2 protects against hypoxic nerve injury by regulating mitophagy through SESN2/AMPK pathway
Source: Front Mol Biosci. 2023 Sep 21;10:1266243. doi: 10.3389/fmolb.2023.1266243 (PMC10551140; doi:10.3389/fmolb.2023.1266243)

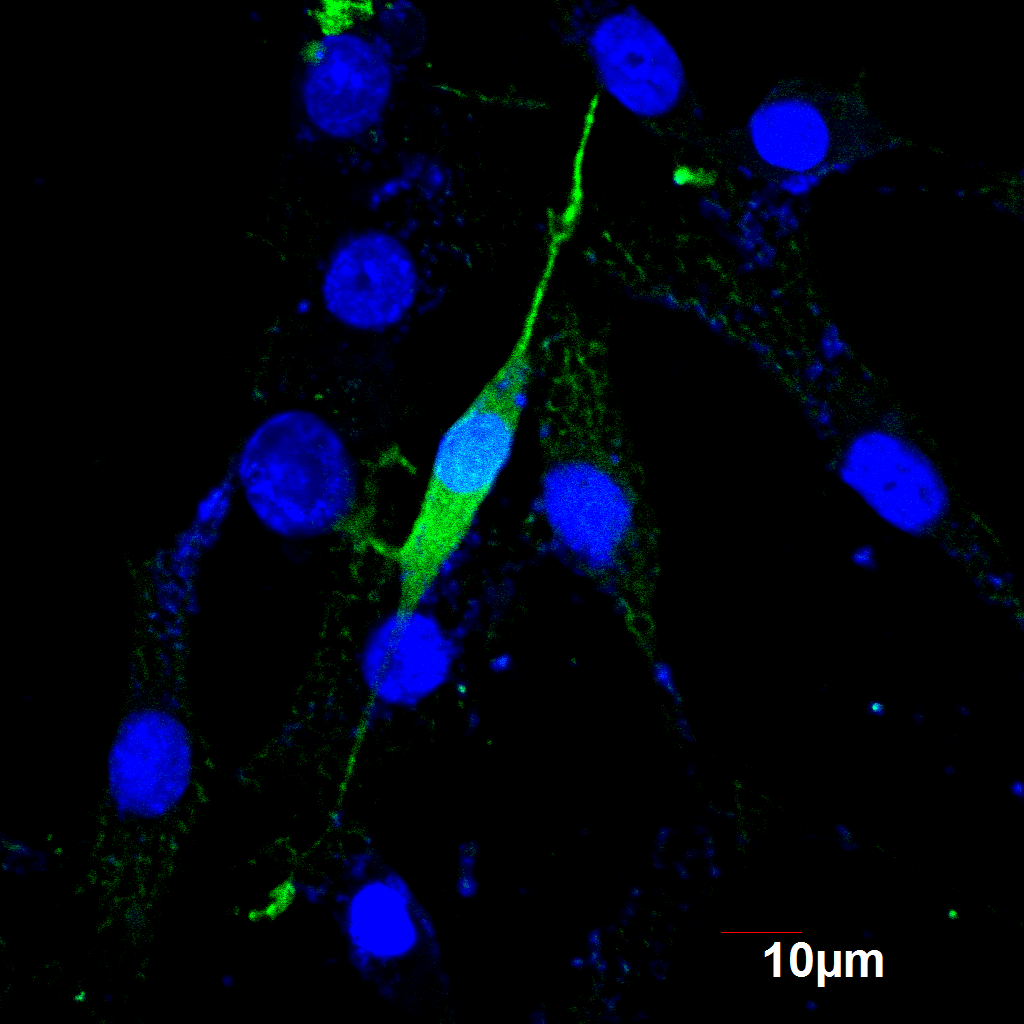

Supplement: Supplementary file 1 [file DataSheet1.ZIP › HC.tif]

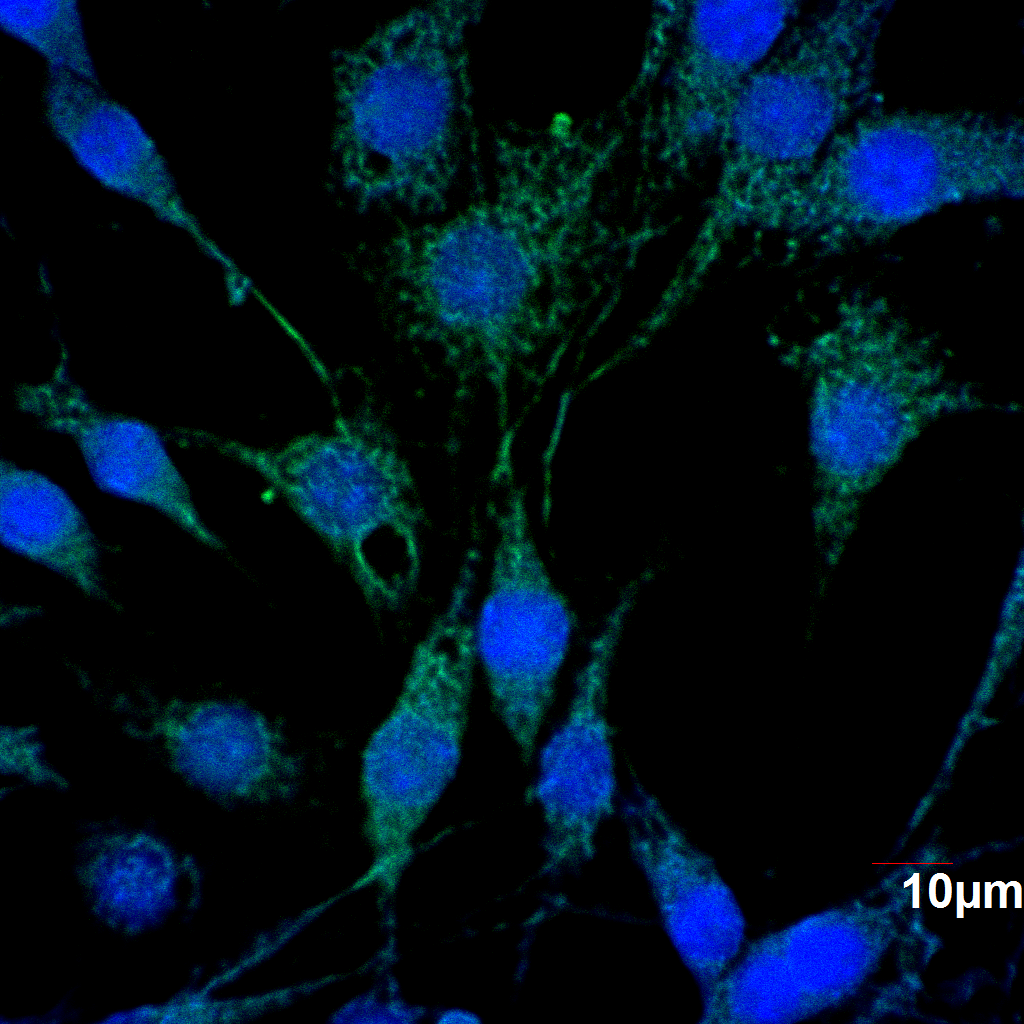

Supplement: Supplementary file 1 [file DataSheet1.ZIP › NC.tif]

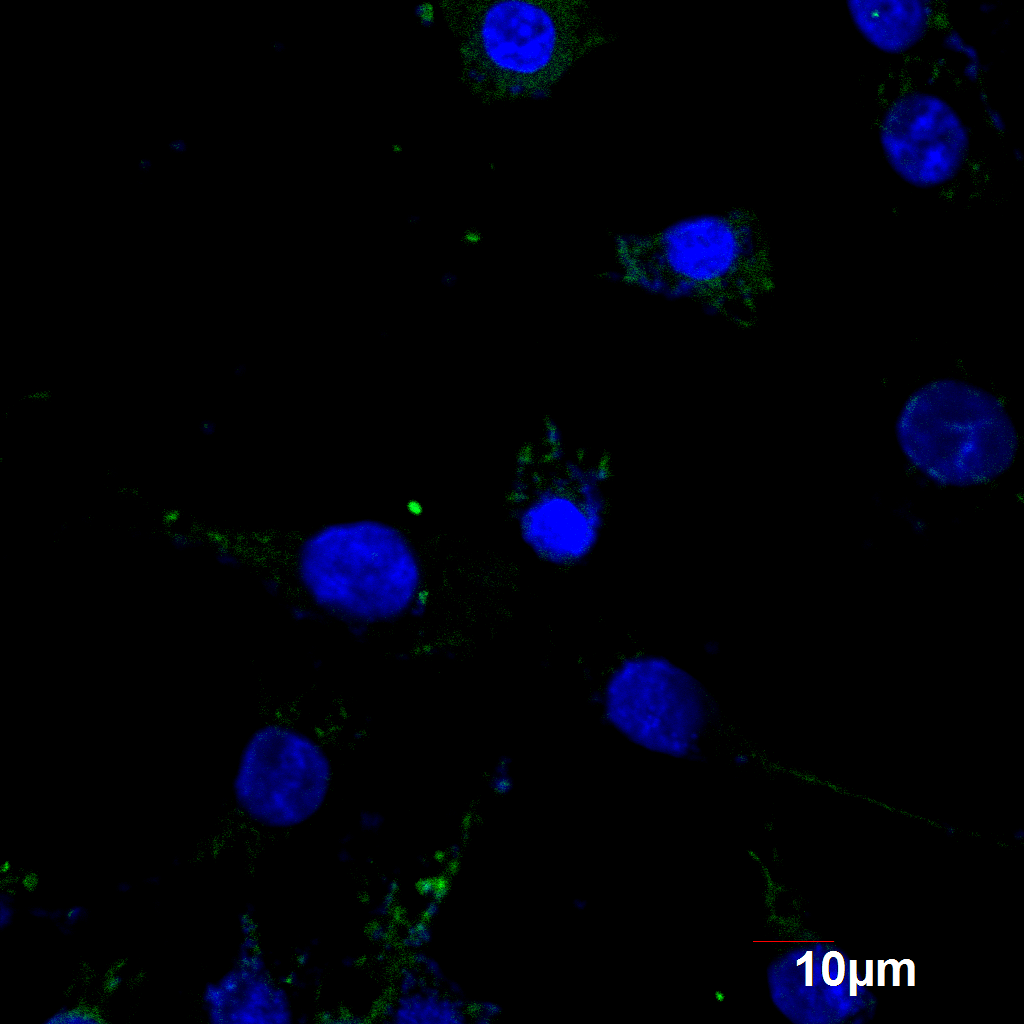

Supplement: Supplementary file 1 [file DataSheet1.ZIP › HC+3MA.tif]
